# Recognition of Human Lower Limb Motion and Muscle Fatigue Status Using a Wearable FES-sEMG System

**DOI:** 10.3390/s24072377

**Published:** 2024-04-08

**Authors:** Wenbo Zhang, Ziqian Bai, Pengfei Yan, Hongwei Liu, Li Shao

**Affiliations:** School of System Design and Intelligent Manufacturing, Southern University of Science and Technology, Shenzhen 518055, China12232626@mail.sustech.edu.cn (P.Y.); shaol@sustech.edu.cn (L.S.)

**Keywords:** functional electrical stimulation, surface electromyography, human motion recognition, muscle fatigue status

## Abstract

Functional electrical stimulation (FES) devices are widely employed for clinical treatment, rehabilitation, and sports training. However, existing FES devices are inadequate in terms of wearability and cannot recognize a user’s intention to move or muscle fatigue. These issues impede the user’s ability to incorporate FES devices into their daily life. In response to these issues, this paper introduces a novel wearable FES system based on customized textile electrodes. The system is driven by surface electromyography (sEMG) movement intention. A parallel structured deep learning model based on a wearable FES device is used, which enables the identification of both the type of motion and muscle fatigue status without being affected by electrical stimulation. Five subjects took part in an experiment to test the proposed system, and the results showed that our method achieved a high level of accuracy for lower limb motion recognition and muscle fatigue status detection. The preliminary results presented here prove the effectiveness of the novel wearable FES system in terms of recognizing lower limb motions and muscle fatigue status.

## 1. Introduction

Functional electrical stimulation (FES) is used to stimulate specific motor neurons using low-frequency pulsed signals to induce muscle movements or simulate normal functional movement [1]. In the field of rehabilitation, FES has been widely used to treat and rehabilitate patients with muscle movement disorders caused by various diseases, including muscle weakness, stroke, hemiplegia, and joint mobility disorders [2,3,4,5]. Recent studies have also demonstrated the value of FES in the field of sports and fitness; for example, FES technology has been widely used by athletes and healthy subjects to develop muscles, increase explosive strength and endurance, and eliminate post-training fatigue [6]. Compared with conventional muscle-strength training methods, FES yields faster and better results [7].

Numerous studies have shown that the efficacy of FES arises from neuroplastic alterations in neural motor circuits, thus indicating the significance of active engagement by the subject during training. FES-based rehabilitation should, therefore, be prolonged and consistent, and the user needs to actively participate in the rehabilitation process during their daily life [8]. Moreover, in real-life scenarios, users often perform full-body exercises with a certain intensity during rehabilitation and training, such as rehabilitation and muscle-strengthening exercises, walking, jumping, aerobics, running, etc. Hence, the essential requirements for such auxiliary equipment include wearability and flexibility [9].

However, existing FES systems [10,11,12,13,14], such as the Myocycle Pro FES Cycling Therapy System created by MYOLYN.LLC, are passive open-loop control systems administered by professional physiotherapists; these devices output stimulus currents in predefined fixed patterns and cannot adaptively adjust the mode of electrical stimulation according to the user’s intention to move [15]. Such systems have several limitations and safety issues, meaning that they are hard to use for daily activities. Issues such as accelerated muscle fatigue, muscle spasms, and even falls and injuries may also occur [16]. Furthermore, these devices are usually bulky and uncomfortable and are not portable, which limits their usability.

In summary, existing FES systems need to be improved from two aspects: (1) not only do they need greater adjustability, based on sensing the user’s state and modulating the mode of electrical stimulation output according to the user’s different motions, but they also need the ability to stimulate muscle fatigue perception and to identify muscle fatigue in order to avoid muscle damage; (2) such systems should be small and lightweight, with good wearability and flexibility, to make them suitable for use in different scenarios such as rehabilitation, training, and relaxation.

This study describes a wearable FES system that uses customized textile electrodes. The textile electrode designed and developed in this study consists of conductive fabric, fabric filler, tie legs, and metal fasteners. The conductive fabric was knitted using Ag/AgCl conductive yarns. The sponge was filled into the conductive fabric as the interlining of the textile electrode. The textile electrodes are breathable, flexible, and stretchable, which can better fit the 3D body shape and reduce skin irritation compared with the traditional gel electrode. The textile electrode is more suitable for long-term monitoring of physical signals and has advantages over the conventional gel electrodes for wearable sEMG devices. The proposed system integrates textile electrodes and surface electromyography (sEMG) sensors to collect sEMG signals. sEMG is important not only for the development of FES methods but also for prosthetic control architectures [17]. Based on the collected signal data, a method is developed for identifying human motions and recognizing muscle fatigue status using deep learning technology. The system presented here is designed for the lower limbs, specifically the calf. Furthermore, the system is driven by voluntary sEMG signals. We note that this system is developed for patients and individuals who can consciously control their muscles in terms of contracting and relaxing them and is not suitable for patients with complete lower limb paralysis.

The main contributions of our work are as follows:

- Recognition of human lower limb motions and muscle fatigue status is achieved by analyzing the sEMG signals collected from the wearable FES device.
- An algorithm for recognizing the motion of the lower limb and the muscle fatigue status is developed based on a parallel deep neural network.

This paper is organized as follows. Section 2 reviews related work. Section 3 describes the architecture of the proposed system. Section 4 introduces our method. Section 5 describes the experiment and the collected data. Section 6 provides an evaluation of the system and discusses the experimental results. Finally, Section 7 concludes the work and outlines future work.

## 2. Related Work

In this work, we develop a wearable FES system based on textile electrodes to collect sEMG signals. The system can recognize lower limb motions and muscle fatigue status using the collected data. A review of related work is given below.

### 2.1. FES-sEMG System

In existing research, FES is combined with sEMG to drive FES and regulate the intensity and cycle of stimulation pulses. Through comparative analyses with existing studies (as shown in Table 1), we found that current studies have several drawbacks: (1) most existing FES-sEMG systems rely on gel electrodes, which are typically disposable and can cause skin irritation and allergies in some individuals with prolonged use, meaning that they are unsuitable for long-term use; (2) current methods for driving an FES system using sEMG signals are relatively simple in existing research, and most studies have focused on perceiving whether the user’s target muscle group is activated based on the changes in sEMG signal features, without identifying the user’s specific motion intention; and (3) existing FES system research is not focused on identifying muscle fatigue status based on sEMG signals.

A prior study [23] reported that the use of textile electrodes was a promising approach to addressing drawback (1); therefore, we designed an FES-sEMG system based on textile electrodes. To tackle drawbacks (2) and (3), we collected sEMG signals using the constructed system and investigated methods for the recognition of human motion and muscle fatigue status based on the acquired signals.

### 2.2. Recognition of Human Motion and Muscle Fatigue Status Recognition Based on sEMG

sEMG is an important physiological electrical signal that can reflect a human’s intention to move. The efficiency of FES can be improved by detecting this intention and using sEMG signals to control the FES, making it a more practical technology.

Following years of academic development, various machine-learning-based classification algorithms have become relatively mature and suitable for use in human motion recognition using sEMG. Saeed et al. used linear discriminant analysis (LDA) and artificial neural network (ANN) classifiers for hand gesture recognition [24] and were able to recognize 11 different gestures using six-channel sEMG signals. In [25], several time-domain features were combined with LDA and used for four gesture recognition tasks. Abdulhamit [26] achieved good classification results for six gesture classification tasks by extracting the energy features of sEMG signals using wavelet packet decomposition and classifying them using a rotation forest classifier. In [27], five types of walking gait (level, uphill, downhill, upstairs, and downstairs) were recognized using sEMG and an IMU combined with a BP artificial neural network. Some studies on human action recognition used deep-learning-based sEMG approaches. Zhang et al. achieved better classification performance compared with LDA under electrode shift by extracting the time–frequency domain features of EMG signals and inputting them into a CNN [28]. Ameri et al. proposed a method based on transfer learning and a CNN to alleviate the impact of electrode shift on the recognition accuracy of wrist action [29]. Yamanoi et al. used a short-time Fourier transform to generate frequency feature maps of sEMG signals for gesture classification [30]. Gautam et al. proposed a method based on transfer learning, CNN, and LSTM for the classification of lower limb motions and prediction of the corresponding knee joint angles [31]. Lee et al. created a wearable textile sensor to measure sEMG signals from the lower limb and used a CNN to classify eight lower limb motions [32].

Machine learning and deep learning methods are commonly used in research on muscle fatigue recognition. Researchers identified three different states of fatigue during Pilates rehabilitation exercises by fusing sEMG and ECG signals using LDA, SVM, and KNN classifiers [33]. The authors of [34] used a genetic algorithm (GA) for sEMG signal feature selection and combined it with an SVM classifier to recognize muscle fatigue and non-fatigue states. Moniri et al. extracted five features from sEMG signals, namely MNF, MDF, RMS, iEMG, and ZC, from 14 different muscles of the human torso [30], and employed a CNN to predict the occurrence of muscle fatigue 25 s in advance. A CNN-SVM method was developed that used RMS, iEMG, MF, MPF, and BSE as inputs to achieve accurate recognition of muscle fatigue status [35]. A recurrent neural network (RNN) was designed for muscle fatigue recognition by extracting nine time-domain and frequency-domain features of sEMG signals [36]. A deep learning model based on long short-term memory (LSTM) was proposed for muscle fatigue recognition [37]. The model employed an enhanced wavelet packet threshold denoising algorithm for sEMG signal denoising, as well as time-domain and frequency-domain feature extraction.

However, traditional machine learning methods are limited in terms of their accuracy and robustness in recognizing lower limb motions and muscle fatigue status. Several studies found that the recognition accuracy of machine learning algorithms drops dramatically when electrode displacement occurs during practical use. Machine learning methods also require manual feature extraction and selection, which is time-consuming and laborious. Although deep learning methods can solve the above problems, most current models have too many layers, which can lead to overfitting during training and low recognition accuracy for sEMG signal analysis. Furthermore, the large number of layers in the network means that high computational power is required for deployment, making them difficult to use in wearable devices. In addition, different input features and network models are often used in current research to recognize motion and muscle fatigue status separately. This approach has certain drawbacks for FES-sEMG systems: (1) the use of two separate models may cause redundancy at the feature extraction stage of the sEMG signals; (2) when the data change, both models need to be retrained, and the parameters of the two models cannot be adjusted simultaneously; and (3) for wearable devices, it is challenging to meet the computational requirements of the two models that output the motion and muscle fatigue status separately.

To address the above issues, we designed a lightweight parallel-structured deep learning model that can fully exploit the spatiotemporal features of sEMG signals to output motion and muscle fatigue status simultaneously. Current deep learning-based methods can rarely detect lower limb motions and muscle fatigue status at the same time.

## 3. System Architecture

**Figure 1:**
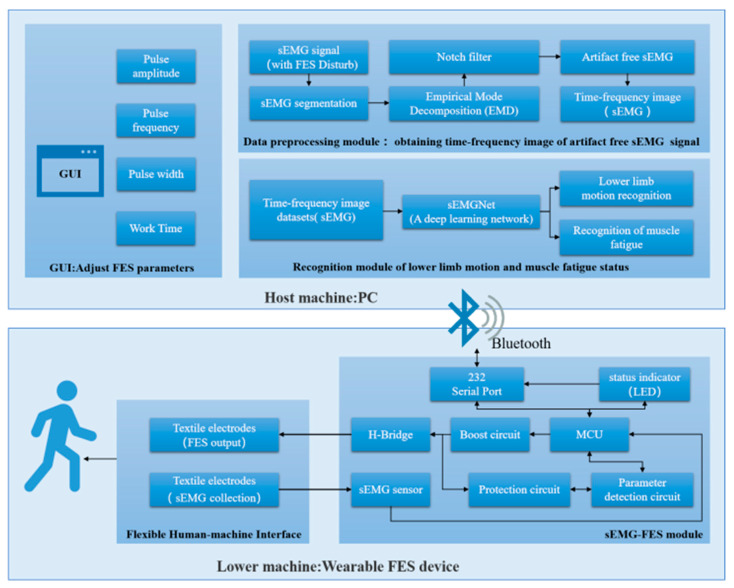
Architecture of the FES system.

In this section, we give a detailed description of the developed wearable FES system. Figure 1 shows a functional diagram of the system, which consists of two main parts: the lower machine and the host machine.

### 3.1. Lower Machine

The lower machine is the wearable FES device, which consists of two modules: the flexible human–machine interface module and the sEMG-FES module.

**Figure 2:**
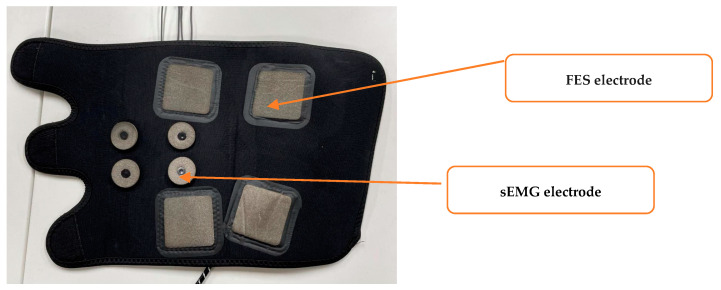
The flexible human–machine interface module.

The flexible human–machine interface module includes textile FES electrodes and textile sEMG electrodes for the left and right legs. These are flexible, dry electrodes based on conductive yarns incorporated into textile materials. The textile electrodes adopted in this study consisted of conductive textiles, textile padding, leg straps, and metal fasteners. Ag/AgCl conductive yarn was used to prepare the conductive textile materials, and a highly absorbent sponge was used as the textile filler, which was placed into the conducting textile to prepare the electrodes. As shown in Figure 2, the 5 × 5 cm square textile electrodes are the FES electrodes. They are placed on the gastrocnemius muscle of the calf, and the size of the textile electrodes is similar to that of the gel electrodes commonly used in FES. The rounded textile electrodes are the sEMG electrodes with a diameter of 10 mm and a center-to-center spacing of 20 mm for the anterior tibialis and gastrocnemius muscles of the calf. We referred to several previous studies to determine the size and placement of the new sEMG electrodes [38].

The sEMG-FES module was developed to acquire sEMG signals and generate FES stimulation pulses. The module included a microcontroller unit (MCU), a boost circuit, an H-bridge output circuit, sEMG sensors, parameter detection circuits, protection circuits, and a Bluetooth module. The whole module measures 4 ×7 cm, making it small and lightweight. Its maximum sampling rate was 8 kHz for sEMG data acquisition, and it offered independent four-channel constant voltage FES output capability. It could simultaneously output two-body symmetric waves with adjustable parameters and had a frequency tuning range of 0–100 Hz and a resolution of 1 Hz. The pulse width was tuned to the range of 0–1000 ms, with a resolution of 10 ms. The excitation amplitude was tuned to the range of 0–70 V, with a resolution of 0.5 V.

### 3.2. Host Machine

The host machine was a PC-based system that included a GUI control interface, a data preprocessing module, and a recognition module for lower limb motion and muscle fatigue status. It was used to control the on/off state, output mode, and output parameters of the FES device.

The GUI control interface was used for the initial configuration of the FES system parameters, including the output voltage, frequency, pulse width, stimulation duration, and other settings.

The data preprocessing module segmented the sEMG signals collected by the lower machine in the presence of FES interference, extracted the sEMG signals without electrical stimulation artifacts, and converted the sEMG signals into time–frequency domain image data.

The recognition module used the time–frequency domain image data of the sEMG signals and the FES-sEMGNet deep learning network to recognize the user’s lower limb motion intention and muscle fatigue status. Monitoring the user’s motion and muscle fatigue in real time allowed the FES output parameters to be better adjusted to improve the effectiveness of rehabilitation training and prevent user injury.

When in use, the PC was connected to the FES device through Bluetooth. The user set the initial FES parameters using the GUI and sent them to the lower machine via Bluetooth. The microcontroller unit in the lower machine configured the boost circuit and the H-bridge output circuit to output a stimulation current with certain parameter settings, such as the amplitude, frequency, pulse width, and training duration. Simultaneously, the parameter detection circuit detected the parameter settings of the output pulses, and the sEMG acquisition circuit was combined with the ADC of the microcontroller unit to capture real-time sEMG signal data from the stimulated leg muscles. These data were communicated in real time to the host machine. The recognition module performed intention recognition calculations based on the preprocessed data, which enabled the user’s own voluntary sEMG signals to drive the FES system.

## 4. Proposed Method

**Figure 3:**
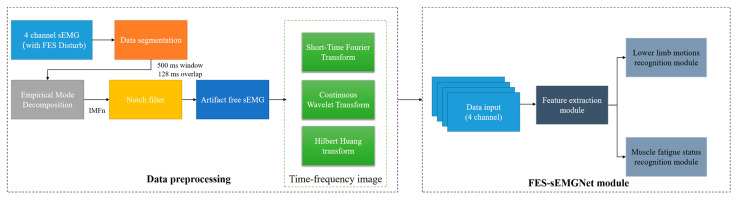
Workflow of the proposed method.

We developed a method to recognize the user’s lower limb motion and muscle fatigue status for use with the wearable FES system, as shown in Figure 3. This involved a data preprocessing flow and an FES-sEMGNet module.

### 4.1. Data Preprocessing

The data preprocessing step included data segmentation, filtering and noise reduction, sEMG image transformation, and division of the data into training and testing sets. First, the raw data were preprocessed using a windowing analysis, where the sEMG signals were segmented into multiple windows using overlapping time-sliding windows.

To eliminate the interference between the sEMG signals of the FES device, a method inspired by Pilkar’s research was adopted to filter out the FES artifacts [39]. In this study, empirical mode decomposition (EMD) and a notch filter were combined to remove the electrical stimulation artifacts from the collected sEMG signals and to extract the voluntary sEMG signals.

Previous studies demonstrated that deep learning techniques can replace traditional methods that are dependent on specific feature designs [28,40]. These techniques directly extract useful information from raw sEMG signals for applications such as motion recognition.

Existing research shows that transforming sEMG signals into time–frequency images and inputting them into CNN networks can improve recognition accuracy [41]. In previous studies, it was demonstrated that transforming sEMG signals into time–frequency images can be used for limb motion recognition [42], detection of neuromuscular diseases [43], recognition of static muscle fatigue [44,45], and muscle fatigue recognition during periodic dynamic contraction processes [46].

To obtain time–frequency images, three commonly used time–frequency transformation methods from existing studies were considered, as described below.

#### 4.1.1. Short-Time Fourier Transform

The short-time Fourier transform (STFT) is a signal processing technique that allows analysis of the time-varying frequency content of a signal. It involves dividing a long signal into shorter segments, applying a Fourier transform to each segment, and then concatenating the results to form a time–frequency representation of the signal. The STFT of a signal $x\left(t\right)$ is given by

(1) $$X(t,f)=\int_{-\infty }^{\infty }x(\tau )w(t-\tau )e^{-j2\pi f\tau }d\tau$$

where x(τ) is the original sEMG signal, and $X\left(t,f\right)$ is the STFT of x(τ) at time t and frequency f. The magnitude of the square of the STFT, $|X(t,f){|}^{2}$, is called the spectrogram and represents the distribution of the signal energy over the joint time–frequency plane [47].

#### 4.1.2. Continuous Wavelet Transform

The continuous wavelet transform (CWT) is a signal processing technique that can be used to analyze non-stationary signals in the time–frequency domain. A signal is convolved with a family of wavelets, which are scaled and translated versions of a mother wavelet, to obtain a time–frequency representation of the signal. The wavelet is defined as follows:

(2) $$\psi_{s,\tau }(t)=\frac{1}{\sqrt{s}}\psi \left(\frac{t-\tau }{s}\right)s,\tau \in R,s>0$$

where $\psi_{s,\tau }(t)$ is the wavelet function, which is also known as the mother wavelet. The mother wavelet is complex conjugate and dilated by the scale parameter s and translated by the translation parameter τ. Conservation of energy is denoted by $\frac{1}{\sqrt{s}}$.

The continuous wavelet transform is defined as follows:

(3) $$W_{x}^{\psi }(\tau ,s)=\langle x(t),\psi_{s,\tau }\rangle =\frac{1}{\sqrt{s}}\int_{-\infty }^{\infty }x(t)\psi^{*}\left(\frac{t-\tau }{s}\right)\mathrm{dt}$$

where $\psi^{*}$ is a complex conjugate of ψ, and $W_{x}^{\psi }(\tau ,s)$ represents the output time–frequency domain image.

Compared with STFT, the CWT provides higher resolution in both time and frequency, but requires more computational resources owing to its continuous nature. In this study, following the work in [48], the bump wavelet was selected as the mother wavelet function.

#### 4.1.3. Hilbert Huang Transform

The Hilbert Huang transform (HHT) is an adaptive time–frequency analysis method that is used to analyze the frequency characteristics of nonlinear and non-stationary signals. It consists of two fundamental steps: EMD and the Hilbert transform (HT). At the data preprocessing stage of our study, in order to eliminate the interference between the FES equipment and the sEMG signals, the EMD algorithm was used to decompose the sEMG signals into multiple IMFs to remove electrical stimulation artifacts. The EMD algorithm decomposes the signal into several IMFs and then applies the HT to each IMF to obtain the analytical signal [39]. Finally, the amplitude spectra of each IMF’s analytical signal are summed to obtain the HHT spectrum. The HT is a mathematical operation that can be used to transform a real signal into a complex signal and is defined as follows:

(4) $$\hat{x}(t)=H[x(t)]=\frac{1}{\pi }\int_{-\infty }^{+\infty }\frac{x(\tau )}{t-\tau }d\tau$$

where $H[x\left(t\right)]$ is the HT of x(t), x(t) is a real signal, and t is time.

Hilbert transformation can convert signals in the time and frequency domains to obtain instantaneous frequency and amplitude information. Specifically, for a real signal x(t), its analytical signal z(t) is obtained as follows:

(5) $$z(t)=x(t)+j\hat{x}(t)=A(t)e^{j\theta (t)}$$

where j is the imaginary unit, $\hat{x}(t)$ is the HT of x(t), a(t) is the instantaneous amplitude of the analytical signal, and θ(t) is the instantaneous phase of the analytical signal, which is defined as follows:

(6) $$a(t)=\sqrt{x^{2}(t)+\hat{x}(t)}$$

(7) $$\theta (t)=\arctan\frac{\hat{x}(t)}{x(t)}$$

Similarly, the instantaneous frequency ω(t) is the derivative of the instantaneous phase θ(t):

(8) $$\omega (t)=\frac{d\theta (t)}{dt}$$

After performing an HT on each IMF component of the sEMG, a Hilbert spectrum, H(ω,t), is constructed by placing a(t) at the appropriate position (ω(t),t) in the time–frequency domain plane, and the sEMG signal x(t) is defined as follows:

(9) $$x(t)=\sum_{j=1}^{n}a_{j}(t)\exp\left(i\int \omega_{j}(t)dt\right)$$

### 4.2. Recognition of Human Lower Limb Motion and Muscle Fatigue Status Using an FES-sEMGNet Model

Deep learning is a machine learning method that can be applied to solve complex tasks using deep neural networks. We combined the obtained sEMG time–frequency images with deep learning techniques to recognize the user’s lower limb motion and muscle fatigue status. The proposed network architecture employs CNN and LSTM networks for feature extraction, which eliminates the need for manual feature selection and extraction, thereby enhancing the efficiency and accuracy of sEMG intent recognition.

#### 4.2.1. Convolutional Neural Networks

CNNs are a class of deep learning algorithms that are extensively employed for computer vision tasks, such as image and video recognition. CNNs are designed to automatically learn and extract relevant features from raw data inputs, rendering them highly effective for image classification, object detection, and segmentation tasks.

A CNN typically comprises several layers, including an input layer, a convolutional layer, a pooling layer, a fully connected layer, and an output layer. A CNN consists of several computational neuron nodes, which establish connections among various structural layers, thus facilitating the transfer of data across the network. The convolutional layer is responsible for extracting features from the input data, while the pooling layer serves to reduce the spatial dimensions of the data. Ultimately, the obtained sample features are mapped onto the feature space, and the fully connected layer employs the extracted features to recognize the input data. In summary, we can harness a CNN to extract features from the input data.

#### 4.2.2. Long Short-Term Memory Networks

RNNs are a class of neural network models that are widely applied to tasks such as natural language processing and time-series analysis. Unlike CNNs, which are feedforward neural networks, RNNs include feedback connections, which allow information to propagate from one time step to the next. This enables an RNN to model sequence data rather than individual data points. LSTM is a variant of RNN that is widely used for processing sequence data [49]. In an LSTM, the issues of vanishing and exploding gradients that arise in traditional RNNs are effectively addressed by incorporating additional gates to control the flow of information, thus facilitating the handling of long sequence data.

Previous works demonstrated the effectiveness of CNNs when applied to sEMG time–frequency images for human motion recognition tasks. Related studies also showed that the capacity of LSTM networks to process temporal information can be exploited to investigate the muscle fatigue status of a user based on sEMG data. As previously mentioned, the primary objectives of this study were to develop a wearable FES system, to enhance the perception and regulation capabilities of an FES system, and to improve its wearability and safety. Therefore, the algorithmic component needs to recognize lower limb motion and muscle fatigue status. Since the algorithm proposed here is intended to be embedded into the MCU of a wearable FES system, its computational complexity should not be excessive, and its computational efficiency must be relatively high.

Based on these considerations, we developed the FES-sEMGNet deep learning model. This model can recognize six lower limb motions and the muscle fatigue status for three rehabilitation exercises.

**Figure 4:**
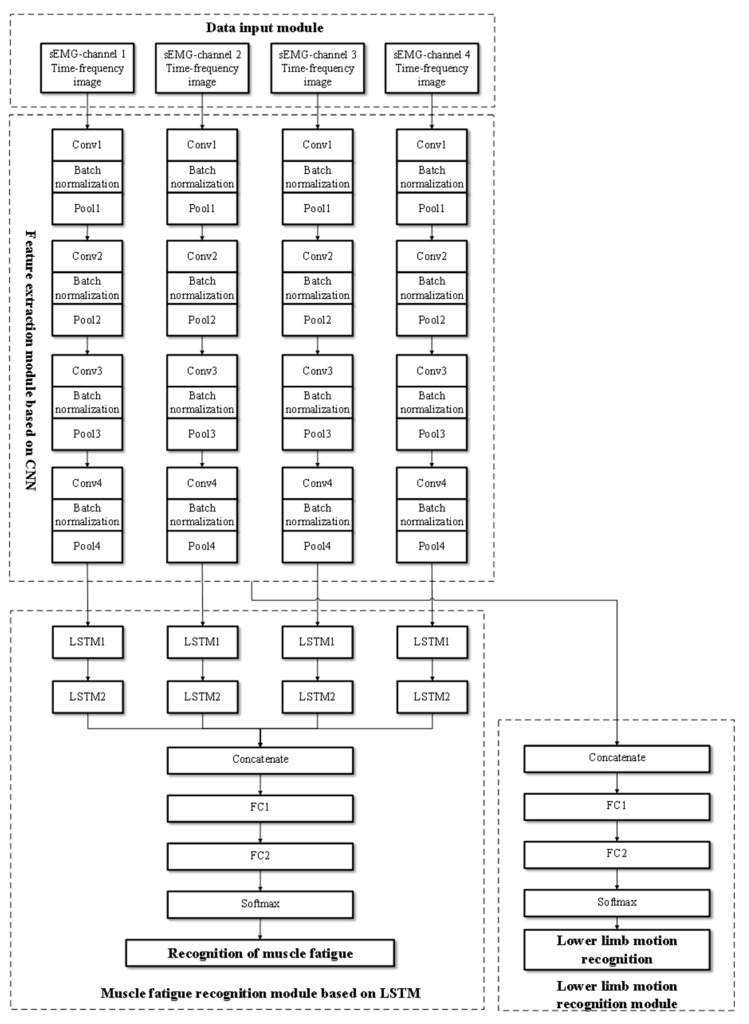
Structure of the proposed FES-sEMGNet model.

Figure 4 shows the network architecture of the proposed FES-sEMGNet model. It is a hybrid model that includes CNN and LSTM components, a data input module, a feature extraction module, a lower limb motion recognition module, and a muscle fatigue recognition module. FES-sEMGNet accepts sEMG time–frequency image data as input, which are initially processed by a CNN-based feature extraction module. The extracted features are then fed separately into a lower limb motion recognition module, with feature fusion layers and fully connected layers, and an LSTM-based muscle fatigue recognition module. Finally, the recognition results for the lower limb motion and muscle fatigue status are output.

The CNN-based feature extraction module consists of four parallel simplified CNNs. Each CNN consists of four convolutional layers and four pooling layers for feature extraction. Batch normalization operations are performed between each convolutional and pooling layer. Following the four pooling layers, a dropout layer is connected, and ReLU is employed as the activation function. This network structure is designed to extract features from the time–frequency images of sEMG signals. The lower limb motion recognition module comprises a feature fusion layer, a fully connected layer, and a Softmax recognition layer. In contrast, the LSTM-based lower limb muscle fatigue recognition module features four parallel networks, each of which includes two LSTM layers connected by linear activation functions, followed by a feature fusion layer and two fully connected layers. This module recognizes lower limb muscle fatigue states based on features extracted from sEMG signals. Its specific network layers and relevant input/output parameters are presented in Table 2.

## 5. Experiment and Data

### 5.1. FES-sEMG Data Acquisition Experiments

#### 5.1.1. Subjects

The subjects in the trial were five healthy adult males with no known neuromuscular impairment, who had abstained from any form of strenuous exercise for 24 h prior to the start of the experiment. The average age was 24 ± 3 years, the average weight was 70.5 ± 5 kg, and the average height was 175 ± 6 cm. This experiment was approved by the Southern University of Science and Technology Medical Ethics Committee (2022PES149). Written informed consent was obtained from the subjects to ensure that all individuals understood the experimental objectives and procedures.

#### 5.1.2. Experimental Process and Data Acquisition

**Figure 5:**
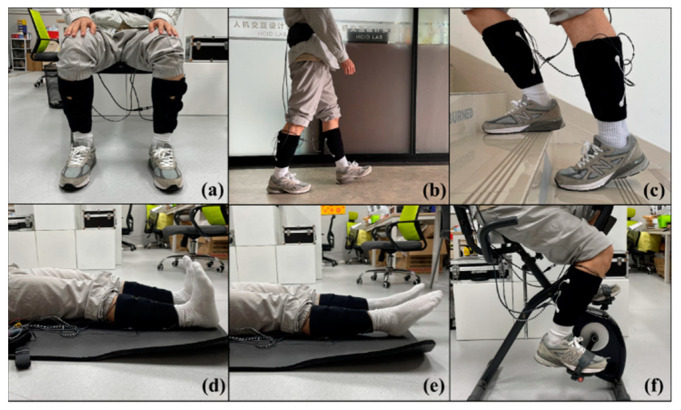
Six lower limb motions: (**a**) sitting, (**b**) walking, (**c**) climbing stairs, (**d**) ankle dorsiflexion, (**e**) ankle plantarflexion, and (**f**) cycling.

To evaluate the lower limb motion intention and muscle fatigue perception capabilities of the proposed wearable FES system, the experiments were divided into daily tasks and fatigue tasks. The daily tasks included three daily activities (sitting, walking, and climbing stairs), while the fatigue tasks included three rehabilitation exercises (ankle dorsiflexion, ankle plantarflexion, and cycling) (Figure 5). Subjects were required to wear the FES system and to complete both experimental tasks while receiving electrical stimulation.

Before the experiment, the skin of the subject’s leg was cleaned with 75% alcohol. This process removed surface debris and grease and was carried out to improve the quality of signal acquisition. The FES system was then worn on the subject’s lower leg, with the textile electrodes in contact with the surfaces of the tibialis anterior and medial gastrocnemius muscles. The FES system was set to a 20 Hz output with a 300 µs pulse width [50], which are commonly used parameters for rehabilitation. The subjects self-adjusted the stimulus amplitude to produce visible muscle contractions and relaxations. Acquisition of the sEMG signal was performed at 8 kHz.

To allow us to collect motion data for the daily tasks, subjects were instructed to repeat each motion in three sets, with each set lasting 5 min. To avoid the influence of muscle fatigue, a rest period of 15 min was provided between sets and activities.

For the fatigue tasks, the subjects self-assessed their fatigue status using the Borg Rating of Perceived Exertion (RPE) scale (Table 3) [51,52]. Previous research established a correlation between the ratings of this scale and fatigue status [53]. Levels 0 to 3 were defined as ‘No feeling of fatigue,’ levels 4 to 6 as ‘Medium fatigue,’ and levels 7 to 10 as ‘Extreme fatigue.’ Before the experiment, the RPE scale was explained to the subjects. During the experiment, subjects were instructed to perform three sets of each motion, with at least 30 min of rest between sets and motions to ensure recovery from muscle fatigue. Each motion was performed consistently, with subjects continuously assessing their fatigue status using the RPE scale. Subjects notified researchers when their self-assessment reached levels 4 and 7. After reaching level 7, subjects maintained the experiment for three more minutes before termination.

Before the experiment, subjects were shown instructional images of the relevant postures, which allowed them to practice and familiarize themselves with the processes.

### 5.2. sEMG Time–Frequency Image Data

**Figure 6:**
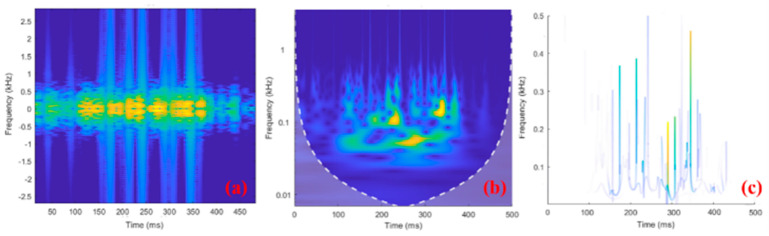
Examples of 500−ms−long sEMG time–frequency images using (**a**) STFT, (**b**) CWT, and (**c**) HHT. In the time-frequency diagram, brighter colors indicate more signal components here.

Data preprocessing (data segmentation, electrical stimulation artifact filtering, and construction of sEMG time–frequency image data) was carried out using functions in MATLAB in offline mode. The input sEMG time–frequency images for the network were unified to 256 × 256 × 3. The raw sEMG signal with a duration of 500 ms was separately converted into a time–frequency image, and the conversion results for the three methods considered here are shown in Figure 6.

To balance the different data types in the dataset, 10,000 images/channel of sEMG time–frequency images were kept for each time–frequency image generation method. They were randomly divided into training, testing, and validation sets using a ratio of 8:1:1.

### 5.3. Training

The design, training, and evaluation of the network model were conducted on a 64-bit Ubuntu operating system, using an Intel 12th-generation Core i7 processor @ 5.0 GHz and 64 GB memory, with a GeForce RTX™ 3080 GPU. Training and evaluation were performed using the PyTorch 1.10 framework. For training of the model, the number of epochs was set to 100, the batch size was set to 50, the dropout parameter was set to 0.3, and the Adam optimizer was used with a default learning rate of 0.001. Since the FES-sEMGNet model needs to complete two tasks, the loss was calculated separately for each task and then summed to give the final loss function, as shown below:

(10) $$\mathrm{loss}(t)={\mathrm{loss}}_{1}(t)+{\mathrm{loss}}_{2}(t)$$

### 5.4. Performance Evaluation

The performance of our FES-sEMGNet model was evaluated based on the numerical results and measured by the precision, recall, F1 score, accuracy, and confusion matrix. The following formulae were used to calculate these statistical indicators:

(11) $$\mathrm{Precision}=\frac{TP}{TP+FP}$$

(12) $$\mathrm{Recall}=\frac{TP}{TP+FN}$$

(13) $$F1\;\mathrm{score}=2\times \frac{\;\mathrm{Precision}\;\times \;\mathrm{Recall}\;}{\;\mathrm{Precision}\;+\;\mathrm{Recall}\;}$$

(14) $$\mathrm{Accuracy}=\frac{TP+TN}{TP+FP+FN+TN}\times 100$$

where TP, TN, FP, and FN represent the true positive, true negative, false positive, and false negative rates for the FES-sEMGNet, respectively.

## 6. Results and Discussion

### 6.1. Discussion of User Comfort and Acceptance of the Implementation of Wearable FES Systems

As mentioned above, in terms of hardware design, the aim of the proposed system was to address the issues of electrodes causing skin allergies and the insufficient wearability of conventional FES devices. In this study, we employed skin-friendly, breathable textile electrodes to address the problem of electrode sensitization as an alternative to conventional gel electrodes. To tackle the shortcomings in terms of usability, 3D cutting techniques were used to fabricate textile electrode-based leggings as flexible human–machine interfaces. A miniaturized sEMG-FES module was developed to reduce the overall size and weight of the system. During the experiments, the subjects reported that wearing the FES-sEMG system was easy and comfortable. The system did not interfere with aerobic exercise throughout the test period, and no cases of skin allergy were observed. However, in these experiments, the sEMG-FES module and power supply were placed at the waist and were connected to the lower leg by wires. This design lacks flexibility and may pose a risk of wire disconnection during intense activities. Future work should consider integrating the sEMG-FES module and power supply into a subject’s leggings or shoes to facilitate user acceptance and applications for rehabilitation training or exercise.

### 6.2. Lower Limb Motion and Muscle Fatigue Status Recognition Performance Based on Different sEMG Time–Frequency Image Inputs

In this subsection, we discuss the performance of the FES-sEMGNet model for lower limb motion and muscle fatigue status recognition based on sEMG time–frequency images obtained using the STFT, CWT, and HHT methods. Three types of sEMG time–frequency image data, generated using the STFT, CWT, and HHT methods, were used as inputs to the FES-sEMGNet network. The goal was to recognize the lower limb motion and muscle fatigue status by assessing the performance of various time–frequency images as network feature inputs. This evaluation enabled the selection of the optimal time–frequency image to serve as the input feature for the FES-sEMGNet network.

For the lower limb motion recognition task, the values for the accuracy of the STFT, CWT, and HHT methods were 91.57%, 92.13%, and 93.33%, respectively. The results for the precision, recall, and F1 score for each of the six motions are shown in Table 4. For the muscle fatigue status recognition task, the values for the accuracy of the STFT, CWT, and HHT methods were 84.50%, 83.15%, and 89.02%, respectively. The results for the precision, recall, and F1 score for muscle fatigue status are shown in Table 5.

As can be seen from Table 4, the accuracy results for the HHT time–frequency images surpassed those of STFT and CWT in the experiments on the lower limb motion recognition accuracy, with the STFT and CWT methods exhibiting similar accuracy. The values for the F1 score for the HHT were 95.02%, 94.28%, 92.08%, 92.41%, 91.39%, and 94.67% for sitting, walking, climbing stairs, ankle dorsiflexion, ankle plantarflexion, and cycling, respectively, which were higher than for STFT and CWT. As illustrated in Table 5, the HHT time–frequency images also yielded the highest accuracy when used as model input in the experiments on muscle fatigue status recognition using the three types of data. The STFT and CWT time–frequency images yielded significantly worse results than the HHT time–frequency images. The F1 score for HHT was consistently higher than for STFT and CWT in all three stages of muscle fatigue status. For example, HHT achieved an F1 score of 91.10% in the ‘No feeling of fatigue’ phase, while the values for STFT and CWT were 85.35% and 87.02%.

These experimental results reveal that the proposed FES-sEMGNet model with HHT time–frequency images outperformed the models with STFT and CWT time–frequency images in the lower limb motion and muscle fatigue status recognition tasks. The HHT time–frequency images yielded significantly better accuracy, precision, recall, and F1 scores for muscle fatigue status recognition compared with their STFT and CWT counterparts. This superior performance can be ascribed to HHT’s capability to extract features with higher temporal and frequency resolution from sEMG signals compared with STFT and CWT. Therefore, the HHT time–frequency images were chosen as input data for the FES-sEMGNet network.

We also observed that the recognition accuracy of muscle fatigue status was marginally lower than for the lower limb motion recognition task. The primary reason for this is that the information related to muscle fatigue is embedded within the temporal information of the sEMG signals. Although we applied the filtering and noise reduction methods described above to eliminate FES interference, FES disrupts the temporality of sEMG signals to some extent. In the future, better techniques for sEMG signals extraction will be needed, particularly under conditions of FES interference.

### 6.3. Comparison with Machine Learning Methods: Lower Limb Motion Recognition

In this study, the input data consisted of 2D sEMG time–frequency images. Table 6 lists the four most commonly used time–domain features (TDFs) for sEMG signal motion recognition. Table 7 presents a comparison of the average recognition accuracies for all subjects during the various motions involved: sitting, walking, climbing stairs, ankle dorsiflexion, ankle plantarflexion, and cycling. For this comparison, we used the HHT spectrogram features and four TDFs with both the proposed method and the LDA classifier. The LDA classifier and four TDFs were selected owing to the widespread use of the LDA classifier in sEMG signal-based classification research. Recent studies demonstrated that the LDA, in combination with these four TDFs, can achieve a high level of motion recognition accuracy from sEMG data [54].

When the LDA classifier was combined with MAV, WL, ZC, and SCC features, it achieved a recognition accuracy of 87.08% for lower limb motion. In contrast, the proposed recognition method (HHT-FES-sEMGNet) achieved a recognition accuracy of 93.33%, which was significantly better than the 4TDFs-LDA method. As shown in Table 7, our HHT-FES-sEMGNet method obtained F1 scores of 95.02%, 94.28%, and 94.67% for sitting, walking, and cycling, respectively. The F1 scores for climbing stairs, ankle dorsiflexion, and ankle plantarflexion were 92.08%, 92.41%, and 91.39%; i.e., slightly lower than those for sitting, walking, and cycling. The 4TDFs-LDA method yielded F1 scores of 88.66%, 88.44%, 86.29%, 85.15%, 88.37%, and 88.50% for sitting, walking, climbing stairs, ankle dorsiflexion, ankle plantarflexion, and cycling, respectively, which were all lower than for the HHT-FES-sEMGNet model. It can also be seen that the F1 scores for climbing stairs, ankle dorsiflexion, and ankle plantarflexion were slightly lower than those for sitting, walking, and cycling.

Table 8 shows the confusion matrices for both methods, which represent the recognition accuracy of the tested samples for the following motions: sitting, walking, climbing stairs, ankle dorsiflexion, ankle plantarflexion, and cycling. The bold diagonal elements of the confusion matrix represent the exact recognition of the general motion categories by the proposed method and the 4TDFs-LDA method, while the other elements represent failures of recognition. The confusion matrix reveals that the slightly lower recognition accuracy for climbing stairs, ankle dorsiflexion, and ankle plantarflexion is due to some of the motions of climbing stairs being misclassified as ankle plantarflexion some ankle dorsiflexion and ankle plantarflexion motions being mutually misjudged. For example, in the HHT-FES-sEMGNet recognition results for ankle dorsiflexion, 3.2% were misclassified as ankle plantarflexion, and for climbing stairs, 2.6% were identified as ankle plantarflexion and 1.9% as ankle dorsiflexion. This is because the muscle contraction patterns for these three motions are relatively similar, particularly for ankle dorsiflexion and ankle plantarflexion.

Based on the above experimental results, it can be concluded that the proposed method yields superior results compared with traditional machine learning and existing approaches and that it is effective for lower limb motion classification tasks.

### 6.4. Comparison with Machine Learning Methods: Recognition of Muscle Fatigue Status

Table 6 lists four time-frequency features (TFFs) that are commonly used for recognizing muscle fatigue from sEMG signals. Table 9 presents a comparison of the average recognition accuracy for the muscle fatigue status of all subjects, for the categories of no fatigue sensation, moderate fatigue, and extreme fatigue. The results from the proposed method are compared with those from the LDA classifier using HHT spectral features and the four TFFs. These four TFFs were selected because numerous related studies on sEMG muscle fatigue recognition showed that they have a high correlation with muscle fatigue status.

When the RMS, IEMG, MF, and MPF features were used in conjunction with an LDA classifier, an accuracy of 83.87% was achieved for muscle fatigue status. In contrast, our proposed method, HHT-FES-sEMGNet, achieved an accuracy of 89.02% for the muscle fatigue status, significantly outperforming the 4TFFs-LDA method. As shown in Table 9, the F1 scores for our HHT-FES-sEMGNet for the categories of no feeling of fatigue, medium fatigue, and extreme fatigue were 91.10%, 88.38%, and 87.56%, respectively; in comparison, the F1 scores for the 4TFFs-LDA method were 86.16%, 82.49%, and 82.95%, which were all lower than for HHT-FES-sEMGNet. Both methods had slightly lower F1 scores for the categories of moderate and extreme fatigue compared with no feeling of fatigue.

Table 10 shows the confusion matrices for both methods. The accurate recognition of the test samples by the proposed method is confirmed across the three fatigue levels: no feeling of fatigue, medium fatigue, and extreme fatigue. Bold diagonal elements in the confusion matrix indicate the accurate recognition of known muscle status categories by the proposed method and the 4 TFFs-LDA method, while other elements indicate recognition failures.

From the confusion matrices, it can be seen that the F1 scores for the medium and extreme fatigue status are slightly lower than for the relaxed status. The primary reason for this is the small number of misclassifications between the first two categories. This effect may be related to the participants’ subjective judgments of fatigue status based on the Borg CR10 scale. Medium and extreme fatigue status are two degrees of fatigue with some subjective bias, but they still fall within a reasonable range.

A comparison with the 4 TFFs-LDA method and an analysis of the other parameters show that the proposed method achieves better results than traditional handcrafted feature methods. This confirms the effectiveness of the proposed method for use in muscle fatigue status recognition tasks.

## 7. Conclusions and Future Work

This study has proposed an sEMG signal-based motion intention-driven wearable FES system. The system employs a textile-based strap and has a lightweight design that offers convenience, minimal weight, and comfort during wear. This can facilitate rehabilitation and training for users in real-life scenarios. When sEMG signals were collected, data preprocessing was performed, and deep learning techniques were utilized. We carried out recognition of lower limb motion and muscle fatigue status using time–frequency images of sEMG signals without manual feature extraction, even under FES. Independent use of an FES system in daily life can improve rehabilitation training outcomes to some extent. The lower limb motion recognition module presented in this study can enable the automatic adjustment of FES output parameters based on the different movements of the user. In addition, muscle fatigue recognition can help to address the issue of FES-induced muscle fatigue, thus enhancing the safety of FES system usage. A combination of these two aspects can promote the independent use of FES devices by users in their daily lives. To the best of our knowledge, this work is the first attempt to combine deep learning techniques with sEMG signals as part of a wearable FES system. It also represents the first implementation of a learning model for the simultaneous recognition of lower limb motion and muscle fatigue status.

A dataset of sEMG signals was constructed using a wearable FES system. This dataset included six motions performed by five healthy subjects under three muscle fatigue conditions and was used for lower limb motion and muscle fatigue status recognition under electrical stimulation. We developed our FES-sEMGNet deep learning model based on the use of sEMG time–frequency images. This lightweight and parallel structured deep learning model simultaneously outputs the motion and muscle fatigue status for driving FES device parameters using sEMG signals. The proposed model eliminates the need for manually designed features and achieves an accuracy of 93.33% for lower limb motion recognition and 89.02% for muscle fatigue status recognition. This is a notable performance improvement compared with conventional methods using manually crafted features. Our experimental results substantiate the effectiveness of the proposed approach and highlight its potential for practical application in wearable FES systems for lower limb motion and muscle fatigue status recognition.

In future work, we plan to conduct clinical trials to validate the practical rehabilitation effects of the proposed motion intention-driven wearable FES system. We expect this system to yield significant clinical outcomes and to assist users in real-life rehabilitation and training settings. We aim to deploy the recognition model on edge computing devices such as FES devices and smartphones. This would free it from its dependence on the laboratory environment, thus enabling real-time operation on resource-constrained platforms.

## Figures and Tables

**Table 1:** Comparison of existing FES-sEMG system studies.

Main Work	Advantages/Disadvantages	Reference
A wearable FES system for lower limb rehabilitation.	The user’s target muscle groups were judged to be activated or not based on changes in sEMG signal characteristics, without identifying the user’s specific motor intention.	[8]
A wireless multi-channel EMG/FES integrated system for upper limb stroke rehabilitation.	The FES-SEMG system used gel electrodes, which can cause skin irritation and sensitization in some people and are not suitable for long-term use.	[18]
Improvement of balance, gait function, and symmetry in the elderly using sEMG-FES.	This study did not focus on the identification of muscle fatigue states based on sEMG signals.	[19]
Assessment of the therapeutic effect of functional electrical stimulation in stroke patients using sEMG.	This study was not a closed-loop control system based on FES-sEMG.	[20]
Using surface EMG signals to aid stroke rehabilitation.	The rehabilitation equipment used in this study was poorly portable and not suitable for long-term monitoring of treatment.	[21]
Improvement and validation of the low-power rehabilitation system based on the application of the ATC technique to sEMG signals.	The user’s muscle fatigue was not monitored during the experiment.	[22]
Recognition of human lower limb movement and muscle fatigue states by a wearable FES-sEMG system.	The wearable system is more suitable for long-term use and is able to detect muscle fatigue to provide a closed-loop FES-SEMG system.	This work

**Table 2:** FES-sEMGNet model parameters.

Module	Layers	Input Size	Output Size
Feature extraction module	Conv1	256 × 256 × 3	256 × 256 × 20
	Pool1	256 × 256 × 20	128 × 128 × 20
	Conv2	128 × 128 × 20	128 × 128 × 20
	Pool2	128 × 128 × 20	64 × 64 × 20
	Conv3	64 × 64 × 20	64 × 64 × 20
	Pool3	64 × 64 × 20	32 × 32 × 20
	Conv4	32 × 32 × 20	32 × 32 × 20
	Pool4	32 × 32 × 20	16 × 16 × 20
Lower limb motion recognition module	Concatenate	16 × 16 × 20 × 4	
	FC1	20,480	2048
	FC2	2048	6
Muscle fatigue status recognition module	LSTM1	20,480	1024
	LSTM2	1024	1024
	Concatenate	1024 × 4	
	FC1	4096	1024
	FC2	512	3

**Table 3:** The Borg Rating of Perceived Exertion (RPE) scale.

RPE Scale	Body Feeling	Subjective Feeling
0	Very easy	No feeling of fatigue
1	No exertion at all	
2	Extremely light	
3	Very light	
4	Light	Medium fatigue
5	Somewhat hard	
6	Mezzo	
7	Hard (heavy)	Extreme fatigue
8	Very hard	
9	Extremely hard	
10	Maximal exertion	

**Table 4:** Lower limb motion recognition results using three different sEMG image inputs.

Motion	Input Data	Precision	Recall	F1 Score
Sitting	HHT	92.84%	97.30%	95.02%
	STFT	91.42%	95.90%	93.61%
	CWT	92.01%	96.70%	94.30%
Walking	HHT	93.77%	94.80%	94.28%
	STFT	93.69%	92.10%	92.89%
	CWT	91.89%	92.90%	92.39%
Climbing stairs	HHT	91.76%	92.40%	92.08%
	STFT	90.47%	91.30%	90.88%
	CWT	91.66%	91.20%	91.43%
Ankle dorsiflexion	HHT	93.55%	91.30%	92.41%
	STFT	90.72%	89.90%	90.31%
	CWT	92.29%	88.60%	90.41%
Ankle plantarflexion	HHT	92.00%	90.80%	91.39%
	STFT	89.02%	90.00%	89.51%
	CWT	91.09%	91.00%	91.05%
Cycling	HHT	96.09%	93.30%	94.67%
	STFT	95.40%	91.30%	93.31%
	CWT	93.90%	92.40%	93.15%

**Table 5:** Muscle fatigue status recognition results using three different sEMG image inputs.

Muscle Fatigue Status	Input Data	Precision	Recall	F1 Score
No feeling of fatigue	HHT	90.65%	91.56%	91.10%
	STFT	87.28%	83.50%	85.35%
	CWT	88.42%	85.67%	87.02%
Medium fatigue	HHT	88.04%	88.72%	88.38%
	STFT	78.85%	84.11%	81.40%
	CWT	80.38%	85.11%	82.68%
Extreme fatigue	HHT	88.35%	86.78%	87.56%
	STFT	83.79%	81.83%	82.80%
	CWT	85.09%	82.72%	83.89%

**Table 6:** Time-domain features (TDFs) of the four sEMG signals used in this method.

Time-Domain Features	Symbol	Definition of the Feature
Mean Absolute Value	MAV	$\mathrm{MAV}=\frac{1}{N}\sum_{i=1}^{N}\left|X_{i}\right|$
Waveform Length	WL	$WL=\frac{1}{N}\sum_{i=1}^{N-1}\left(X_{i+1}-X_{i}\right)$
Zero Crossing	ZC	$ZC=\sum_{i=1}^{N-1}\left[\left(x_{i}\cdot x_{i+1}<0\right)\cap \left(\left|x_{i}-x_{i+1}\right|>\varepsilon \right)\right]$ *
SlopeSign Change	SSC	$SSC=\sum_{i=2}^{N-1}\left[\left(x_{i}-x_{i-1}\right)\cdot \left(x_{i}-x_{i+1}\right)\right]>\varepsilon$

* $\varepsilon =R*\sqrt{\frac{1}{N}\sum_{j=1}^{N}{\left(x_{NM}[j]\right)}^{2}}$. Following the work in [44], R ranges from 0 to 6 with a step of 0.02. $x_{NM}[j]$ represents samples of the signal at rest. N is the total number of samples.

**Table 7:** Results of lower limb motion recognition with three different sEMG image inputs.

Motion	Input Data	Method	Precision	Recall	F1 Score
Sitting	HHT	FES-sEMGNet	92.84%	97.30%	95.02%
	4 TDFs	LDA	86.71%	90.70%	88.66%
Walking	HHT	FES-sEMGNet	93.77%	94.80%	94.28%
	4 TDFs	LDA	87.03%	89.90%	88.44%
Climbing stairs	HHT	FES-sEMGNet	91.76%	92.40%	92.08%
	4 TDFs	LDA	86.99%	85.60%	86.29%
Ankle dorsiflexion	HHT	FES-sEMGNet	93.55%	91.30%	92.41%
	4 TDFs	LDA	86.65%	83.70%	85.15%
Ankle plantarflexion	HHT	FES-sEMGNet	92.00%	90.80%	91.39%
	4 TDFs	LDA	85.84%	84.90%	85.37%
Cycling	HHT	FES-sEMGNet	96.09%	93.30%	94.67%
	4 TDFs	LDA	89.31%	87.70%	88.50%

**Table 8:** The confusion matrix of HHT-FES-sEMGNet and 4TDFs-LDA for lower limb motion recognition.

**HHT-FES-sEMGNet**						
	**Sitting**	**Walking**	**Climbing stairs**	**Ankle dorsiflexion**	**Ankle plantarflexion**	**Cycling**
**Sitting**	**97.3%**	0.9%	0.6%	0.5%	0.4%	0.3%
**Walking**	2.2%	**94.8%**	2.3%	0.1%	0.1%	0.5%
**Climbing stairs**	0.7%	1.7%	**92.4%**	1.9%	2.6%	0.7%
**Ankle dorsiflexion**	1.7%	0.9%	1.7%	**91.3%**	3.2%	1.2%
**Ankle plantarflexion**	2.4%	1.0%	2.2%	2.5%	**90.8%**	1.1%
**Cycling**	0.5%	1.8%	1.5%	1.3%	1.6%	**93.3%**
**4 TDFs-LDA**						
	**Sitting**	**Walking**	**Climbing stairs**	**Ankle dorsiflexion**	**Ankle plantarflexion**	**Cycling**
**Sitting**	**90.7%**	2.1%	1.9%	2.1%	1.9%	1.3%
**Walking**	2.6%	**89.9%**	2.5%	1.9%	1.4%	1.7%
**Climbing stairs**	2.5%	3.2%	**85.6%**	2.8%	3.1%	2.8%
**Ankle dorsiflexion**	2.9%	3.2%	2.6%	**83.7%**	4.7%	2.9%
**Ankle plantarflexion**	3.7%	1.8%	3.9%	3.9%	**84.9%**	1.8%
**Cycling**	2.2%	3.1%	1.9%	2.2%	2.9%	**87.7%**

**Table 9:** Results of muscle fatigue status recognition with three different sEMG image inputs.

Muscle Fatigue Status	Input Data	Method	Precision	Recall	F1 Score
No feeling of fatigue	HHT	FES-sEMGNet	90.65%	91.56%	91.10%
	4 TFFs	LDA	85.71%	86.61%	86.16%
Medium fatigue	HHT	FES-sEMGNet	88.04%	88.72%	88.38%
	4 TFFs	LDA	82.03%	82.94%	82.49%
Extreme fatigue	HHT	FES-sEMGNet	88.35%	86.78%	87.56%
	4 TFFs	LDA	83.87%	82.06%	82.95%

**Table 10:** The confusion matrix of HHT-FES-sEMGNet and 4TDFs-LDA for muscle fatigue status recognition.

	HHT-FES-sEMGNet			4 TFFs-LDA		
**Ankle dorsiflexion**	No feeling Of fatigue	Medium fatigue	Extreme fatigue	No feeling Of fatigue	Medium fatigue	Extreme fatigue
No feeling of fatigue	**97.3%**	0.9%	0.6%	**87.7%**	5.3%	7.0%
Medium fatigue	2.2%	**94.8%**	2.3%	7.2%	**84.8%**	8.0%
Extreme fatigue	0.7%	1.7%	**92.4%**	8.0%	9.7%	**82.3%**
**Ankle plantarflexion**	No feeling Of fatigue	Medium fatigue	Extreme fatigue	No feeling Of fatigue	Medium fatigue	Extreme fatigue
No feeling of fatigue	**97.3%**	0.9%	0.6%	**88.8%**	7.0%	5.7%
Medium fatigue	2.2%	**94.8%**	2.3%	7.5%	**77.7%**	14.8%
Extreme fatigue	0.7%	1.7%	**92.4%**	6.8%	14.3%	**78.8%**
**Cycling**	No feeling Of fatigue	Medium fatigue	Extreme fatigue	No feeling Of fatigue	Medium fatigue	Extreme fatigue
No feeling of fatigue	**97.3%**	0.9%	0.6%	**86.6%**	7.8%	5.6%
Medium fatigue	2.2%	**94.8%**	2.3%	6.9%	**82.9%**	10.2%
Extreme fatigue	0.7%	1.7%	**92.4%**	7.6%	10.4%	**82.1%**

From the data and analysis presented above, we can see that when the HHT time–frequency images are used with the FES-sEMGNet model, high levels of accuracy, precision, recall, and F1 score are achieved in the recognition tasks for six lower limb motions and three levels of muscle fatigue. It can be concluded that the proposed method is effective in recognizing lower limb motion and muscle fatigue status in the context of FES.

## Data Availability

The data generated or analyzed as part of the research are not publicly available at this moment.
